# Maternal‐to‐Infant Transfer of Medications for Type 2 Diabetes Mellitus Via Breastmilk: A Systematic Review of Available Evidence and Clinical Guidelines

**DOI:** 10.1002/cpt.70211

**Published:** 2026-02-11

**Authors:** Katherine Richardson, Joshua Kiptoo, Beatrice Mpora Odongkara, Francis Williams Ojara, Catriona Waitt

**Affiliations:** ^1^ School of Medicine University of Liverpool Liverpool UK; ^2^ Infectious Diseases Institute Makerere University Kampala Uganda; ^3^ Department of Pharmacy, Faculty of Medicine Mbarara University of Science & Technology Mbarara Uganda; ^4^ Department of Pediatrics & Child Health, Faculty of Medicine Gulu University Gulu Uganda; ^5^ Department of Pharmacology and Therapeutics, Faculty of Medicine Gulu University Gulu Uganda; ^6^ Department of Women’s and Children’s Health, School of Medicine University of Liverpool Liverpool UK

## Abstract

This review evaluates the available pharmacokinetic data on the plasma‐to‐breastmilk transfer of first‐ and second‐line T2DM drugs against available clinical guideline recommendations. A list of drug therapies for treating T2DM was generated from national and international clinical guidelines. A systematic search of research articles reporting human plasma and breastmilk drug concentrations was conducted in Scopus, PubMed, Google Scholar, and LactMed® in accordance with the Preferred Reporting Items for Systematic Reviews and Meta‐Analyses (PRISMA) guidelines. Studies evaluating breastmilk drug transfer in T2DM, with fully accessible abstract and main text reported in English, were included. Study quality was evaluated using the ClinPK checklist. Authors evaluated clinical guideline recommendations on the use of T2DM drugs in lactation and the basis upon which such recommendations were made. Only 5 out of 20 drugs (*metformin*, *glyburide*, *glipizide*, *tolbutamide*, *and semaglutide*) have clinical data on plasma‐to‐breastmilk transfer. Metformin and tolbutamide were detectable in maternal plasma and breastmilk. Half (51.7%) of guideline recommendations provide explicit guidance. Only 4.4% of recommendations were based on clinical evidence. Over half (57.8%) of recommendations were accessible online, and most guideline recommendations (78%) were against the use of antiglycemic agents while breastfeeding. The scarce clinical evidence to guide T2DM drug therapy during breastfeeding available has several design and methodological limitations. Published recommendations remain largely inconsistent, thus perpetuating uncertainty in the use of T2DM drug therapies in lactation. Addressing knowledge gaps is critical in developing clinical consensus to optimize T2DM drug therapy among breastfeeding mothers.

**Study Highlights**

**WHAT IS THE CURRENT KNOWLEDGE ON THE TOPIC?**

Safety information avaiable for medication use in pregnancy and lactataion remains limited for most drugs. Some guidance does exist from NICE, ADA, and RACGP on the use of medication for T2DM in breastfeeding, but it is not clear how strong the evidence base for these is.

**WHAT QUESTION DID THIS STUDY ADDRESS?**

What studies exist to describe how T2DM medications are transferred from maternal plasma to breastmilk, and how robust are these? Which clinical guidelines exist on the use of T2DM medications in breastfeeding?

**WHAT DOES THIS STUDY ADD TO OUR KNOWLEDGE?**

This review shows that the use of T2DM medications in breastfeeding is grossly understudied, leading to mixed guidance from NICE, ADA, and RACGP among other sources about their transfer and safety to the nursing infant.

**HOW MIGHT THIS CHANGE CLINICAL PHARMACOLOGY OR TRANSLATIONAL SCIENCE?**

This review consolidates current studies and guidance on the use of T2DM medications in breast feeding and thereby demonstrates the need for rigorous lactation studies to provide evidence on breastmilk transfer and infant exposure to T2DM drug therapies.

Type 2 diabetes mellitus (T2DM) affects ~500 million adults worldwide.^1^ Compared with healthy individuals, those with T2DM have a twofold higher mortality risk,^2^ and an increased risk of end‐organ damage, including cardiovascular events and kidney failure.^3^ The pharmacotherapy of T2DM reduces risk of disease progression, diabetes‐related complications, morbidity, and mortality.^4^ Despite significant efforts to improve drug labeling for pregnancy and lactation, over 90% of approved medications are still used “*off‐label*”.^5^ Due to significant benefits to both mother and infant,^6^ the World Health Organization (WHO) recommends initiation of breastfeeding within the first hour of birth and exclusive breastfeeding for the first 6 months of life.^7^ Globally, about 40% of babies are exclusively breastfed at 6 months of age.^8^ In the United States, breastfeeding rates decreased from 83.2% to 55.8% between birth and 6 months postpartum, due to concerns about maternal or child health, including concern about infant undernutrition or illness, maternal illness including HIV infection, or the need for medicine.^9^ Only about 10% of clinically approved medications have any information on use in pregnant and lactating women^5^; this gap may be associated with (i) early cessation of breastfeeding, (ii) cessation of maternal medicine, (iii) improper use of medication during breastfeeding, (iv) and/or maternal anxiety due to cessation of breastfeeding or uncertainty due to potential infant safety risk. Over the past three decades (1990–2021), the global contribution of high body mass index (BMI) to T2DM disability‐adjusted life years (DALYs) increased by 24.3%.^1^ Maternal weight gain leading to obesity^10^ is an independent risk for metabolic syndrome, T2DM, and mortality among women of childbearing age.^11^ Breastfeeding has been shown to offer maternal protection against developing T2DM (Hazard ratio, 0.65 [0.49–0.86])^12^ and weight gain.^13^ Despite the maternal and infant benefits, breastfeeding targets remain largely unmet, accounting for an estimated 16% of child mortality annually worldwide.^8^

Over the past decade, novel T2DM medications have been introduced in the market^14^ including Glucagon‐like Peptide‐1 (GLP‐1) receptor agonists like Semaglutide; Sodium Glucose Cotransporter 2 inhibitors (SGLT2i) such as Empagliflozin; and dual GIP/GLP‐1 receptor agonists like Tirzepatide (**Table** **1**).

**Table 1:** A summary of treatment guidelines indicating first‐ and second‐line drugs used in the management of type 2 diabetes mellitus

Treatment guideline	First‐line drugs	Additional therapies
RACGP, 2020^15^	Metformin	*SGLT2‐i, DPP4‐i, SU, GLP‐1 RAs, insulin
ADA guidelines, 2024^4^	Metformin, *SGLT2‐i	TZDs, DPP4‐i, *GLP‐1 RAs, SUs, insulin
NICE guidelines, 2023^16^	Metformin, *SGLT2‐i, DPP4‐i	TZDs (pioglitazone), SUs, Insulin
UCG, 2024^17^	Metformin	SUs (Glimepiride), insulin, SGLT2‐I, DPP4‐I, GLP1‐RAs, TZDs
Combined list of medicines for the management of type 2 diabetes mellitus	Metformin; Dapagliflozin, Empagliflozin, Canagliflozin, Ertugliflozin (*SGLT2‐i); Alogliptin, Vildagliptin, Sitagliptin, Saxagliptin, Linagliptin (DPP‐4 i); Tolbutamide, Glyburide, Glimepiride, Gliclazide, Glipizide (SUs); Semaglutide, Exenatide, Liraglutide, Tirzepatide (*GLP‐1 RAs); Pioglitazone (TZDs)	

ADA, American Diabetes Association; DPP4‐i, Dipeptidyl Peptidase‐4 Inhibitors; GLP‐1 RAs, Glucagon‐like Peptide‐1 Receptor Antagonists; NICE, National Institute for Health and Care Excellence; RACGP, Royal Australian College of General Practitioners; SGLT2‐i, Sodium Glucose Cotransporter Protein 2 Inhibitors; SU, Sulfonylureas; TZDs, Thiazolidinediones; UCG, Uganda Clinical Guidelines.

In addition to their well‐documented glucose lowering efficacy, these newer medications have added benefits in weight management, cardiovascular, and renal protection. Semaglutide gained widespread use for the treatment of T2DM (Ozempic®) and Obesity (Wegovy®) by influencing gastric emptying and energy metabolism.^18^, ^19^ However, no clinical recommendation exists to guide its use in lactation, despite the US FDA guidance to conduct pre‐licensure lactation studies for drugs anticipated to be used in women of childbearing age.^20^ This poses a challenge as the burden of pre‐existing T2DM has doubled in the last two and a half decades^21^ and the inventory of available therapies keeps expanding, yet limited evidence is available to guide their use in lactation. Adoption of novel techniques like physiologically based pharmacokinetic (PBPK) modeling is key to strengthening the existing evidence landscape.^22^ Most clinical treatment guidelines remain “silent” regarding the use of T2DM medication during breastfeeding. Prominent guidelines like the National Institute for Health and Care Excellence (NICE), American Diabetes Association (ADA), and the Royal Australian College of General Practitioners (RACGP) provide some guidance on the use of medication for T2DM in breastfeeding, but it is not clear how strong the evidence base for these is.

The present review aims to collate existing clinical pharmacokinetic data and outline evidence gaps to guide practitioners and breastfeeding mothers on infant exposure to maternal first‐ and second‐line drugs used for the treatment of T2DM.

## METHODS

First, a list of 20 first‐ and second‐line drugs (apart from Insulin) recommended for the treatment of type‐2 diabetes mellitus was generated from the latest published versions of four representative clinical guidelines sources; including ADA, American Academy of Clinical Endocrinology (AACE), NICE, and the Uganda Clinical Guidelines (UCG) (**Table** **1**). Clinical recommendations guiding the use of the 20 drugs among breastfeeding women being treated for T2DM were drawn from regional and internationally representative sources including RACGP, Derbyshire Joint Area Committee (JPC), North East London Guidelines, NICE guidelines – British National Formulary drug summary, and WHO. Additionally, online databases, including the LactMed®, the motherToBaby information service by the Organization of Teratology Information Specialists (OTIS), and the European Medicines Agency (EMA) were reviewed. All clinical guidelines and online information sources were searched for published data until March 2025. For each drug, each guideline source was searched for clinical recommendation and summarized as “Recommended” (Y), Not Recommended (N), suggesting Alternative drug (A); a case‐by‐case risk–benefit decision over the drug’s use in breastfeeding (R); and where sources suggested that inconclusive evidence existed (I). No label was denoted where no clinical recommendation was provided for a particular drug and the guideline was considered “silent” on the drug’s use during breastfeeding. Except where guidelines reported inconclusive evidence (I) or were considered “silent” for any of the 20 drugs, reasons underlining the basis of clinical recommendations were summarized as (1) trialed only in animal studies, (2) Potential risk in nursing infant, (3) limited data/“relevant published data not found as of revision date,” (4) “It is unknown,” (5) reason in relation to pharmacological properties of drug, and (6) due to studies found on transfer of the drug in breastfeeding.

Secondly, a systematic search of published studies was conducted focusing on studies that reported concentrations of first‐ and second‐line antidiabetic drugs (apart from Insulin) in breastmilk and/or infant plasma. Four electronic databases were searched, including Google scholar, Scopus, PubMed, and LactMed using the following search strategy: (Drug) AND (blood OR serum OR plasma OR human) AND (breast) AND (milk) AND (postpart* OR breastfeed* OR lactat) AND (Pharmacokinet). The online databases were searched for published data until March 2025. The databases were searched without time limitation; however, only studies reported with available abstracts, full text and reported in English were considered for the initial screening. Screening of identified articles was done independently by two authors (KR & JK), and two senior authors were involved in adjudicating where disagreements existed. All duplicate entries were removed following manual Microsoft Excel article screening of retrieved articles reporting plasma to breastmilk transfer of any of the drugs.

### Inclusion criteria

Studies considered for initial screening reported individual or mean/average plasma, serum, or breastmilk concentrations of any of the listed drugs for the treatment of T2DM. Included studies were then appraised for quality, considering that the authors explicitly reported the study drug, design, sampling strategy, participant demographics, clinical characteristics, etc., according to the ClinPK checklist.

### Data extraction

The extracted information was summarized (**Table** **2**). All individual and mean plasma and breastmilk concentrations of drugs reported in the individual studies were digitized using WebPlotDigitizer (Version 4.7). Where individual concentrations were reported in different units of concentrations (e.g., mmol/L or mg/L), conversions were made to reflect concentration in ng/mL. Since none of the studies reported substantial infant drug concentrations, such data was not included in the extraction table.

**Table 2:** Characteristics of research articles describing plasma to breastmilk transfer of drugs used to treat type 2 diabetes mellitus

Drug	Dosage	Indication	Sample size	Maternal age/Body weight (years/kg)	Race	Postpartum age (days)	Matrix	Sampling	Analysis	Quantification	Breastmilk transfer	Infant exposure, IDD (RIDD)	ClinPK assessment, % (score)	References
Metformin	500 mg p.o, twice daily	T2DM	7	32.5/111.1	Caucasian	4–17	mDBS, mDBMS	Steady‐state, Single trough/peak (2 hours post‐dose)	Mean concentrations	HPLC‐UV	M: P_Cav_ ratio, 0.63 [0.36–1.00]	< 0.014–0.070 (0.65 [0.43–1.08] %)	66 (14/21)	[23]
	500–3000 mg p.o, 1–3 times daily	T2DM, GDM, PCOS	6	32.5/97.7 ± 18.6	NR	> 90	mDBS, mDBMS	Steady‐state, mDBS (0–24), mDBMS (2–3 hour intervals)	NCA (TOPFIT software), parametric tests	HPLC–MS	M: P_AUC_ ratio [0.40 ± 0.11]	0.13 (0.21%), 0.15 (0.14%), 0.21 (0.21%), and 0.28 (0.43%)	71.4 (15/21)	[24]
	500 mg p.o, twice/once daily,	T2DM & PCOS (multiple dose), Healthy Volunteer (single dose)	3 (multiple dose), 5 (single dose)	38.8/73.3 (multiple dose), 34/64.2 (single dose)	Caucasian	60–420 (multiple dose), 210–435 (single dose)	mDBS, mDBMS	Steady‐state, mDBS/BMS (0–12 hours), with 2 hours. Lag for mDBMS (multiple dose) Extra mDBS sample at 24 hours and mDBMS (2–72 hours) (single dose)	NCA (TOPFIT software)	HPLC	M: P_AUC_ ratios: 0.37, 0.50, 0.71 (multiple dose) 72‐hour M: P_AUC_ ratios: 0.27, 0.47 (Single dose)	0.18, 0.20, 0.21% (multiple dose) 0.11, 0.25% (single dose)	76.2 (16/21)	[25]
	500 mg p.o, three times or once daily (ER)	T2DM, PCOS	7	34/97	Caucasian	150–750	mDBS, mDBMS	Steady‐state, mDBS (0–8 hours), mDBMS (0–24). A single mDBS/BM trough/peak (2 hours. post‐dose)	NCA (TOPFIT software)	HPLC	M: P_AUC_ ratio, 0.34 (95% CI: 0.2–0.5)	0.04 [0.02–0.06](0.28 [0.16–0.4]%)	66.7 (14/21)	[26]
	500 mg p.o, single dose	Healthy voolunteer	1	35/59	Caucasian	210	mDBS, mDBMS	mDBS (0 ‐ 72 hours), mDBMS (4×/day over 72 hours)	NCA (WinNonLin V 5.2, TOPFIT software)	HPLC	Maternal plasma M: P: AUC_0–72hrs_, 0.9; AUC_0,∞_, 1.5	0.3%	70 (14/20)	[27]
Tolbutamide	500 mg p.o, twice daily	T2DM	2	NR	Caucasian	3	mDBS, mDBMS	Steady‐state, single peak concentrations (4 hours post‐dose)	Mean concentrations	NR	Average Serum concentration: 0.35, 0.45 mg/L, Breastmilk concentration: 0.18, 0.03 mg/L	NR	45 (9/20)	[28]
Glyburide	5 mg non‐micronized p.o (single dose) **OR** 5/10 mg p.o (multiple dose)	T2DM	6 [5 mg], 2 [10 mg] (single dose study); 3 (multiple dose)	36/81.7 (single dose), 30/116 (daily dose)	Caucasian	NR (single dose) 6 [5–8] (multiple dose) post cesarean delivery	mDBS, mDBMS	Steady‐state, mDBS/BM (2 ‐ 8 hours) post‐dose (Single dose) Steady‐state, single trough/peak concentrations (multiple dose)	NCA	HPLC	Undetectable in mDBS/BM	< 1.5% (5 mg), < 0.7% (10 mg) (single dose) < 28% (multiple dose). Undetectable in mDBMS	81 (17/21)	[29]
Glipizide	5 mg p.o, daily (IR)	T2DM	2	33/110	Caucasian	11 [7–16] (post cesarean delivery)	mDBS, mDBMS	Steady‐state, single trough/peak 3 hours post‐dose	Theoretical IDD	HPLC	0.20 peak/undetectable trough. Undetectable in mDBMS	< 27% (mean theoretical exposure)	81 (17/21)	[29]
Semaglutide	0.25–2.4 mg SubQ., weekly (mean, 0.5 mg/Week)	Weight loss	8	_/92.8	Caucasian, black, Hispanic	1–2 yours (4 mothers); > 6 months (3 mothers)	mBM	mBM at 0, 12, 24 hours post‐dose	Adjusted RID to account for intramuscular/oral bioavailability. Simulated weekly maternal dosing using Simulx (2024R1)	LC–MS	Undetectable in breastmilk (*C*_average_ and *C*_max_)	Worst‐case scenario analysis considering weekly maternal dosing (0.56 mg) and projected RID_FP_ (< 1.26%) and RID_F_ (< 1.12%)	80 (16/20)	[30]

AUC, area under the curve; C_av_, average drug concentrations; C_peak_, peak drug concentration; ER, extended‐release formulation; GDM, gestational diabetes mellitus; HPLC, high performance liquid chromatography; IDD, infant daily dose (mg/kg/day); IR, immediate release formulation; LCMS, liquid chromatography‐mass spectrometry; M:P, milk‐to‐plasma ratio; mBM, maternal breastmilk; mDBMS, maternal dried breastmilk spots; mDBS, maternal dried blood spots; NCA, non‐compartmental analysis; NR, not reported; PCO, polycystic ovary syndrome; p.o., per oral; RIDD, relative infant daily dose(%); RIDFP, relative infant dose following oral and subcutaneous drug administration; T2DM, type‐2 diabetes mellitus; UV, ultraviolet.

### Outcomes and statistical analysis

The primary outcome in the review was a direct measure of plasma to breastmilk transfer of T2DM drugs, measured as drug concentrations in maternal plasma and/or breastmilk. Pooled analysis of maternal milk to plasma ratios (M:P ratios) of the different drugs was not possible, as five out of eight studies reported individual drug concentration‐time profiles for a single drug (Metformin). A secondary outcome was clinical recommendations on the use of T2DM drugs in lactation, as obtained from clinical guidelines and online databases. All eligible clinical lactation study characteristics were tabulated. Individual and population concentration‐time profiles for plasma and breastmilk were then plotted in Microsoft Excel.

### Quality assessment

The quality of the retrieved pharmacokinetic studies was assessed using the reporting requirements according to the ClinPK checklist,^31^ which is widely used in evaluating reporting of pharmacokinetic studies (**Table** **S1**). Two independent reviewers (KR and JK) assessed the studies independently, after which a senior author was invited to adjudicate where disagreements ensued.

## RESULTS

**Figure 1:**
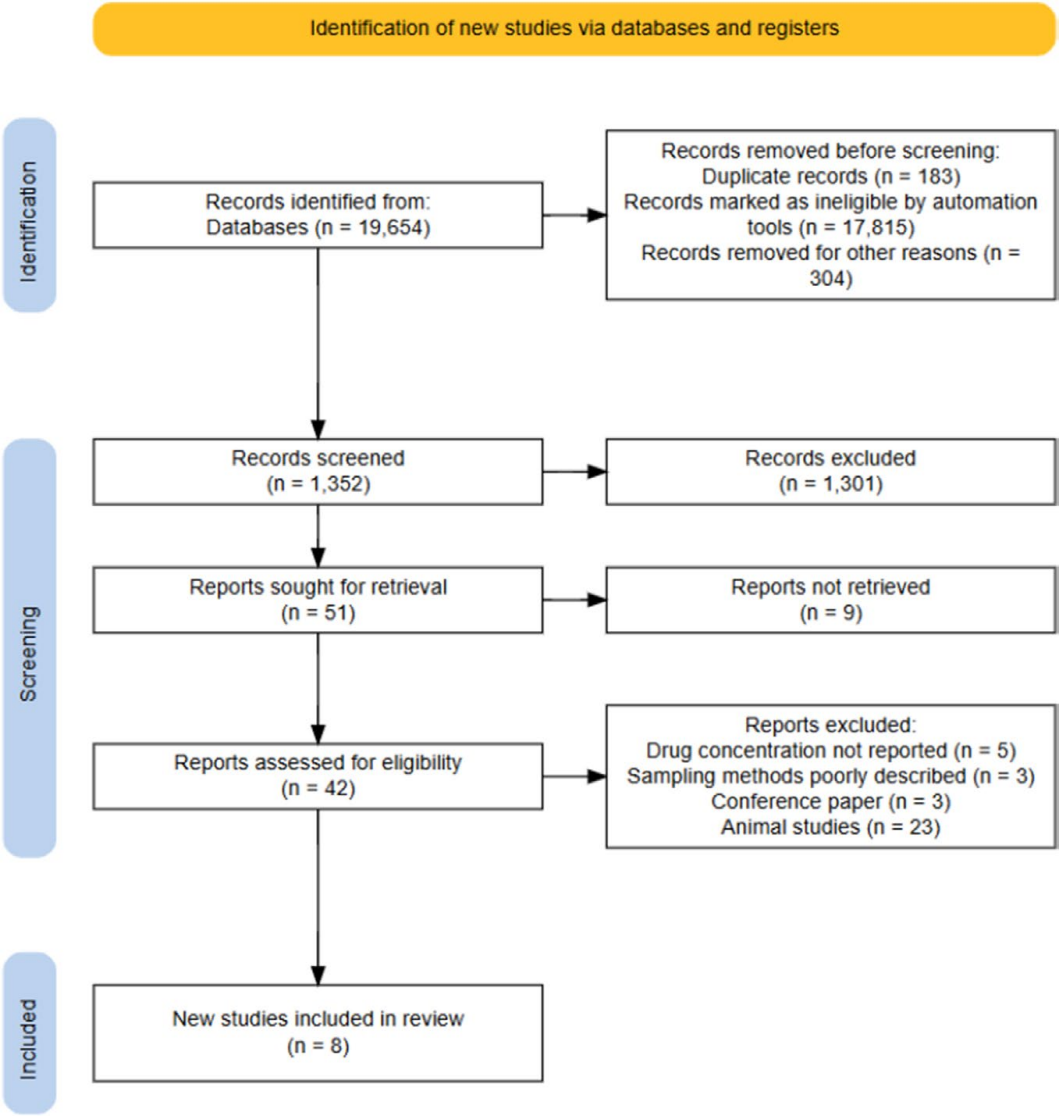
PRISMA‐flowchart showing identification and screening process of published clinical studies evaluating pharmacokinetics of drugs used in type‐2 diabetes mellitus. Plasma to breastmilk transfer of drugs for the treatment of type 2 diabetes mellitus.

A total of 19,654 articles published between 1967 and 2025 were retrieved from the online search. Only a total of 1352 articles were eligible for abstract screening, and 51 articles were eligible for full‐text screening. Out of the 42 articles available for full‐text screening, only 8 were eligible for inclusion in the study (**Figure** **1**). All studies involved a single drug except the study by Feig *et al*.^29^. The eight articles included in the study reported plasma to breastmilk transfer of 5 out of 20 drugs considered in the review, including Metformin, Glyburide (Glibenclamide), Glipizide, Tolbutamide, and Semaglutide. Metformin had the most studies conducted between 1967 and 2008. No study considered in the current review evaluated the effect of the drug on breastmilk production or composition, or the amount of breastmilk consumed based on infant weight before and after breastfeeding. All the studies involved both maternal plasma and breastmilk samples, except for the study by Diab *et al*. (“*milk‐only study*”). Considering the US FDA guidance to industry for lactation studies,^32^ the majority of the included studies conformed to the guidance, although there was significant variability in study sampling design (duration and the postpartum sampling period), determination of M:P ratio, and infant daily dose estimations (**Table** **2**).

### Evaluation of drug transfer into breastmilk

Breastmilk to maternal plasma (M:P) ratios and infant daily dose (IDD) indices are used to evaluate drug transfer and infant exposure. The M:P ratio can be predicted or derived from clinical data and provides an empirical method to compare drug distribution between plasma and breastmilk.^33^ The US FDA recommends M:P ratios based on total exposure (AUC) rather than single timepoint measurements.^20^ The absolute IDD estimates the total drug amount ingested through breastmilk, calculated by multiplying average milk drug concentration by milk volume consumed.^34^ The relative infant dose (RID) compares the calculated IDD to either therapeutic pediatric doses (if licensed for pediatric use) or maternal weight‐adjusted doses (if only licensed for older children/adults). RID provides an understanding of the relative dose ingested by the baby via breastmilk,^35^ and is calculated as:

$$\begin{array}{c} \mathrm{RID}=\left[\text{absolute infant dose}\;\left(\mathrm{mg}/\mathrm{kg}/\mathrm{day}\right)/\text{maternal dose}\right.\\ \left.\;\left(\mathrm{mg}/\mathrm{kg}/\mathrm{day}\right)\right]\;\text{multiplied}\;\mathrm{by}\;100. \end{array}$$

Neither infant dose calculation is able to accurately predict infant safety risk, especially in drugs with non‐dose‐dependent toxicities. These exposure indices are empirical and may not accurately predict actual drug partitioning in breastmilk or ultimate infant safety as they are not reflective of the specific biologic or pharmacokinetic properties of each drug. In practice, most drugs are deemed “safe” for lactation based on M:P ratio less than 1 and/or RID less than 10%. These cut‐offs, however, may lack accuracy as they’re based on expert opinion rather than scientific evidence.

### Metformin

**Figure 2:**
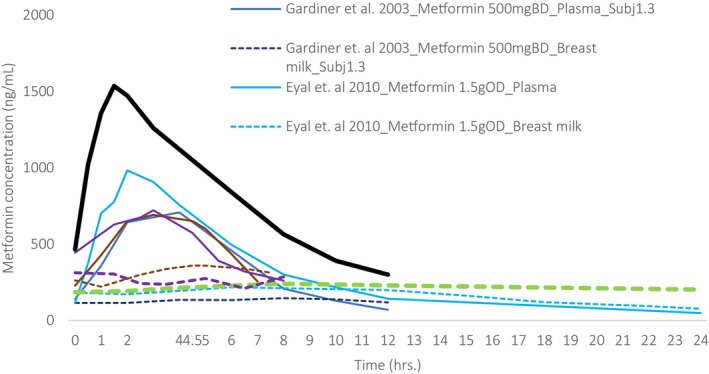
Metformin plasma (solid lines) and breastmilk (dashed lines) concentration–time profiles under steady‐state conditions after multiple doses across five clinical studies. Thin lines: individual profiles and thick lines: mean profiles obtained from several individuals. Data were digitized from the publications using WebPlotDigitizer (Version 4.7).

**Figure 3:**
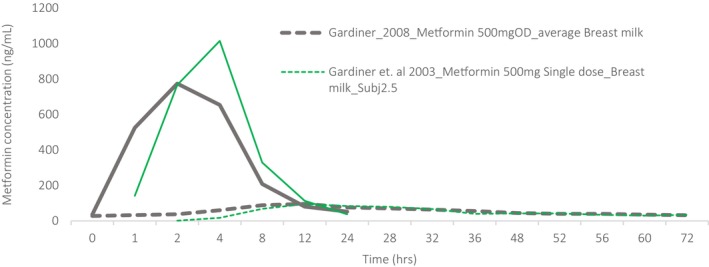
Metformin plasma (solid lines) and breastmilk (dashed lines) concentration‐time profiles after a single dose. Thin lines: individual profiles and thick lines: mean profiles obtained from several individuals. Data were digitized from the publications using WebPlotDigitizer (Version 4.7).

**Figure 4:**
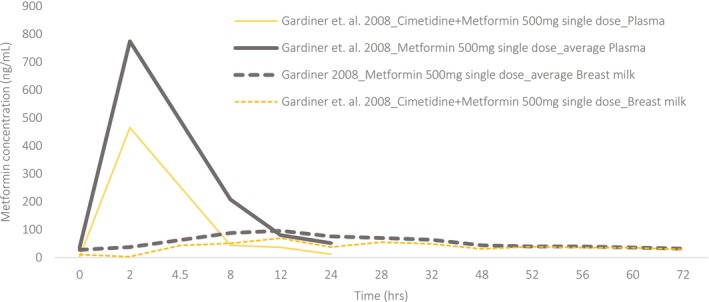
Metformin plasma (solid lines) and breastmilk (dashed lines) concentration‐time profiles with/without co‐administration of cimetidine. Thin lines: individual profiles and thick lines: mean profiles obtained from several individuals. Data were digitized from the publications using WebPlotDigitizer (Version 4.7).

Five studies reported plasma to breastmilk transfer^23^, ^24^, ^25^, ^26^, ^27^ (**Figures** **2**, **3**, **4**), with two other studies evaluating the effect of pregnancy and renal transporters on lactation pharmacokinetics.^24^, ^27^ All studies reported exposure among breastfed infants (0–25 months) of Caucasian mothers treated for T2DM, except a study by Gardiner *et al*. which included a single healthy breastfeeding woman. Under steady‐state conditions, intensive sampling of maternal plasma and breastmilk was conducted over a single dosing interval in four studies. One study analyzed peak and trough maternal plasma and breastmilk concentrations immediately before and 2 hours post‐dose, respectively.^23^ Only Gardiner *et al*.^25^ attempted to quantify drug in infant plasma, which were undetectable. Three studies reported simultaneously determined individual milk and plasma drug concentration‐time profiles, and two Eyal *et al*. and Hale *et al*. reported mean maternal concentration‐time profiles in plasma and breastmilk^24^, ^26^ (**Figure** **2**). Generally, pharmacokinetic analysis was done using non‐compartmental analysis, and infant breastmilk intake was assumed to be 0.15 L/kg/day. The M: P ratio was less than 1^23^; however, higher predictions were observed in drug–drug interaction studies between Cimetidine and Metformin^27^ (**Figure** **4**). Overall, Metformin does not accumulate in breastmilk and is considered “*safe*” in lactation.

Both Glyburide and Glipizide were undetectable in breastmilk despite being present in maternal plasma (glipizide) or undetectable in both compartments (glyburide).^29^ Theoretical RID estimates for glyburide and glipizide were 28% and 27% respectively—both exceeding the FDA’s 10% safety threshold. However, clinical evidence supports the safe use of both agents during breastfeeding, suggesting that despite high theoretical RIDs, actual infant exposure is likely minimal. Semaglutide was undetectable in breastmilk samples from mothers treated for at least 3 weeks. Simulated analysis yielded a theoretical RID of only 1.26% and no reported adverse effects in breastfed infants, confirming its safety profile during lactation.^30^ A single study reported mean serum and breastmilk concentrations of Tolbutamide^28^ (**Table** **2**).

### Assessment of study quality

All eight studies were scored using the “ClinPK” checklist. Three items (items 12, 20, and 21) were deemed irrelevant for clinical lactation studies and were excluded from the analysis as they addressed studies describing population pharmacokinetic covariates models, extracorporeal dialysis parameters, or bioequivalence evaluation. Study conformity to the “ClinPK” checklist consistently met the majority of applicable criteria, with most studies on Metformin scoring at least 21/24 applicable criteria^23^, ^24^, ^25^, ^26^ (**Table** **S12**).

### Clinical guideline evidence on the use of antidiabetic medicines during breastfeeding

**Figure 5:**
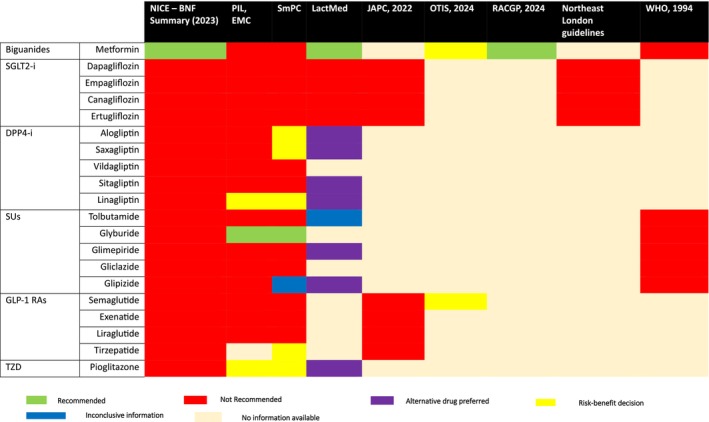
Clinical evidence on use of drugs for T2DM during breastfeeding. DPP4‐1, dipeptidyl peptidase type 4 inhibitors; GLP‐1 RAs, glucagon‐like peptide receptor antagonists; SGLT2‐i, sodium glucose transporter – type 2 inhibitors; SU, sulfonyl ureas; TZDs, thiazolidinediones.^15^, ^16^, ^36^, ^37^, ^38^, ^39^, ^40^, ^41^

For the 20 drugs considered in the review, a total of 180 clinical recommendations were expected across the nine guideline sources (a product of the number of drugs and the clinical guideline sources). 51.7% (93/180) of the recommendations either provided an explicit recommendation on drug use in lactation (50.6%) or acknowledged inconclusive evidence to guide drug use during breastfeeding (1.1%), whereas the rest were “silent” on drug use in lactation. Among guidelines with explicit recommendations, about 78% (71/91) were against drug use in breastfeeding (N), 7.7% (7/91) suggested either use of another drug or risk–benefit discussions, and only 5.5% (5/91) supported use in breastfeeding. Online and electronic databases including LactMed® and the electronic compendium sources accounted for more than half (57.8%, 52/91) of the explicit recommendations sources. Metformin and Glyburide are explicitly recommended for use in breastfeeding in at least one guideline source, based on human clinical evidence. Reasons for recommending against drug use in lactation were consistent across most guidelines, mostly due to “unknown” evidence of infant safety (73.3%) and limited evidence because drug was “trialled in pre‐clinical animals” (15.6%). A small proportion of clinical recommendations were based on evidence from human studies (4.4%). This is not surprising as only five of the 20 drugs recommended as first‐ and second‐line therapies in T2DM had identifiable lactation pharmacokinetic studies (**Figure** **5** **and** **Table** **S2**).

## DISCUSSION

Appropriate labeling is important to ensure safe and effective clinical use of T2DM drug therapies in lactation. Only about one‐quarter (25%) of T2DM therapies have data characterizing breastmilk transfer. Less than 10 percent (4.4%) of recommendations were based on human studies. About half of the guideline recommendations were “silent” regarding the use of antidiabetic drugs during lactation. Whereas this silence is not unique to T2DM, impressive strides have been achieved in other therapeutic areas relevant to lactating women, including psychotropics,^42^, ^43^, ^44^ antibiotics,^45^ anti‐epileptics,^46^ cardiovascular agents,^47^ etcetera.

Understanding infant drug exposure through breastmilk is important for safe and effective clinical use of drugs.^48^ Breastmilk partitioning of metformin was mostly estimated using AUC derived maternal M:P ratios. Comparison of IDD and therapeutic doses is preferred,^20^ but most study comparisons used the maternal weight‐adjusted doses. Whereas it was theoretically determined that Glyburide and Glipizide may not be “*safe*” in lactation (RIDD > 25%),^49^ it could be argued that Metformin, Semaglutide, Glipizide, and Glyburide are generally “*safe”* due to minimal breastmilk transfer and/or undetectable drug concentrations in breastmilk. The discrepancy relates to the bioanalytical assays for the quantification of Glipizide and Glyburide in breastmilk (Lower limit of quantification: 0.08 mcg/mL [CV < 5%: 0.8–2.0 μg/mL]).

Only three out of five studies reported infant safety following potential exposure via breastmilk, of which two studies evaluated infant blood glucose measurement,^25^ in addition to neonatologist assessment and subjective reports from the mother.^26^ Importantly, no infant adverse effects were reported in these studies, which is predictable from the saturable (OCT‐mediated) mammary transfer and low infant oral bioavailability of Metformin.^27^ Notably, no other studies attempted to measure infant exposure based on infant blood plasma, which is far superior to pharmacokinetic modeling and the only way to measure true drug transfer. Infant Metformin exposure via breastmilk is negligible with little variability, making it unnecessary to rely on timing of Metformin dosing relative to breastfeeding as a clinical infant safety risk mitigation measure (**Figure** **2**). Variability in plasma pharmacokinetics is potentially governed by renal transporter polymorphisms and time‐of‐day administration schedules.^50^ A larger study population may address concerns of inter‐individual variability and heterogeneity in study design (**Figure** **3**).^20^ Only one mother in Diab *et al*. reported infant signs of transient diarrhea and loss of appetite, which did not warrant withdrawal as the overall infant exposure period was considered short.

The US FDA and EMA provide regulatory guidelines on how data from clinical lactation studies can inform drug labeling.^51^ These stipulate that “*milk‐plasma*” studies are preferred in cases of suspected drug accumulation in breastmilk. Sample size consideration depends on expected pharmacokinetic variability. Most studies included in this review were “*milk‐plasma*” (maternal)^14^, ^18^, ^19^, ^28^, ^42^, ^48^ with only one “*milk‐only*” study evaluating Semaglutide transfer.^30^ Considerations of the type and timing of milk sampling are critical when evaluating breastmilk partitioning of highly lipophilic, acidic, or basic drugs, and/or drugs that rely on paracellular transport mechanisms. Fewer compositional and quantitative changes are expected in “mature” milk (4–6 weeks postpartum) compared with colostrum.^52^ Within‐day and within‐feed variability has been reported, with higher fat in hind milk (2–3‐fold higher)^53^ and notable circadian variability.^54^ Except for the single dose study by Feig *et al*. (Glyburide),^29^ all studies reported the postpartum period of milk collection (type of milk). However, none reported the timing of milk sampling relative to breastfeeding and the time of day. This may explain the variation in observed milk partitioning and the overall infant exposure, especially for highly lipophilic drugs. Paracellular drug transport is highest in stage II lactogenesis (2–3 days postpartum) allowing for increased plasma to breastmilk transfer. This coincides with the period of increased incidences and severity of infant adverse event reports.^55^ Maternal samples were obtained at steady state, except for the Metformin^27^ and Semaglutide “*milk‐only*” study.^30^ Some of this variability may have been accounted for by accurately capturing the necessary information then using population pharmacokinetic analysis; however, sample sizes per study were too small for this to be feasible.

Semaglutide has unique formulation and pharmacokinetic properties compared with the small molecule drugs discussed in the review, and is given by a weekly subcutaneous injection. US FDA guidelines^20^ recommended appropriate timing of sample collection within the period of peak plasma drug concentrations for such long‐acting drugs. Based on Wegovy® dosing schedule, steady‐state plasma concentrations are attainable in about 16 weeks compared with the shorter exposure duration (averagely 4.6 weeks) in the study.^30^ Semaglutide was not detectable in any of the analyzed breastmilk samples, which may be attributed to poor timing of sample collection (samples collected too soon), contrary to the US FDA recommendation of sample collection within 24‐hours of maximum breastmilk concentrations. The possible mis‐timing of sample collection underscores the need design studies with consideration of pharmacokinetic properties.

Measurement of AUC, which accounts for fluctuation in infant exposure over the sampling time, rather than single timepoint provides a better understanding of overall infant exposure, and the consequential infant safety risk. Measurements of drug in breastmilk over a short period of time may lead to inaccurate conclusions and clinical recommendations. None of the studies evaluated the effects on breastmilk production and composition.^20^ To accurately estimate infant daily milk intake, the US FDA recommends that the expressed breastmilk volume is measured, and the infant weighed before and after breastfeeding. However, most studies estimate an infant milk intake volume (0.15–0.2 L/Kg/day) for practicality. Semaglutide (Ozempic®, Wegovy®) has considerable effects on maternal body weight, gastric emptying, and energy metabolism,^18^, ^19^, ^56^ which may ultimately affect breastmilk composition. Except for the 1967 study, studies included in the present met most of the reporting requirements as per the ClinPK checklist. However, a few checklist requirements were inapplicable to all the retrieved studies, including description of population pharmacokinetic covariates models, extracorporeal dialysis parameters, and drug bioavailability for studies exploring formulation differences. Whereas the ClinPK checklist seems the best available tool to evaluate clinical lactation pharmacokinetic studies, it may not comprehensively assess all quality aspects of clinical studies.

We appraised guideline recommendations and evidence‐based electronic databases intended for both healthcare professionals^21^, ^22^, ^31^, ^32^, ^33^, ^34^, ^35^, ^53^ and patient/care givers. Less than 10% (~4.4%) of clinical recommendations were based on human studies. More than half (57.8%) of recommendations were retrieved from easy to access electronic databases (SmPC, PIL, OTIS, and LactMed®). Recommendations to guide use of the selected drugs during lactation were absent in about half (48.3%) of the guideline sources (**Figure** **5**). This concurs with results from evaluation of all United States. This concurs with results from evaluation of all US FDA approved drugs, with only half of drug labels reporting some information on lactation and up to 43% of the data obtained from preclinical animal research.^5^ We included one international guideline source (WHO) reflecting the needs of more global population. We were unable to include other clinical guidelines as we could neither retrieve any information relevant to the subject of interest (American Diabetes Association, ADA), nor access the guideline at the time of the review (Chinese Diabetes Society guidelines, CDS).^57^ Two guideline sources providing practice guidance in a limited jurisdiction (within the UK) were included. Such guidelines may reflect local needs and practices when treating T2DM in lactating women. Apart from the EU electronic compendium (SmPC and PIL), other electronic recommendation sources included LactMed (established in 2006) and OTIS (established in 1987). Overall, information from electronic databases included in the review is managed by scientific experts in research and health care, regularly updated based on emerging evidence, and is widely referenced by reputable global health stakeholders.^15^, ^56^ However, LactMed is widely preferred for reasons that its expert reviewers synthesize clinical data, provide recommendations and alternative drugs where necessary, and that it has been accessible longer than the MotherToBaby database (started in 2013).^40^ The authors summarize available guideline recommendations and the basis reason(s) upon which such recommendations were made (**Table** **S2**).

Several findings emerged when appraising the adequacy of recommendation sources in supporting decision making on clinical use of antidiabetic drugs in lactation (**Figure** **5**). Firstly, none of the drugs have consistent recommendations across sources. Where recommendations were available, the majority (73%) lacked any form of scientific backing (“*it was unknown*”). Most sources based their recommendation on evidence from preclinical research (rats); however, such evidence is largely inaccurate due to cross‐species physiological differences. Available clinical lactation studies were limited by few study subjects, unrepresentative sampling, and inaccuracy in their analytical methodology. Secondly, many sources are inconsistent. The WHO guidelines (1994) state that “*Both sulfonylureas and biguanides should not be used during pregnancy and breastfeeding*…”, which contradicts information in the WHO model list of drugs (2002) and the SmPC which supports the use of Glyburide in breastfeeding albeit with caution.^36^, ^37^ Again, some guidelines like the ADA (2024) remark that “*Most diabetes medications*, *including insulin and metformin*…” when summarizing recommendations for consideration. Umbrella descriptions like “*Most diabetes medications*” are misleading and dangerous as they irrationally presume extremes of safety possibilities especially for newer drugs like SGLT2 inhibitors where no single lactation study has been conducted. The third observation is that sources often failed to offer an assertive decision of whether a drug was safe in breastfeeding. For example, the SmPC often recommended a risk/benefit decision with a doctor to be made, allowing for inconsistent judgments and decisions to be made by separate doctors, depending on what guideline is used. LactMed recommended alternative drugs or suggested that certain drugs would be preferred over others in their class as opposed to offering concrete guidance. This allows for interpretative differences which may result in inconsistent and unsafe clinical decisions. Furthermore, options are limited when choosing an “alternative drug” that “may be preferred” with no drug being recommended for use across all nine sources. This means a clinician would have to actively go against at least one of the source recommendations. Guidelines provided are often confusing with some sources (EMC) not explicitly stating any risk to the newborns infant whilst recommending that infants have glucose monitoring throughout such that “*breastfeeding seems to be compatible*, *but as a precautious measure monitoring of the fully breastfed infant’s blood sugar level is advisable*.” NICE guidelines were found to be the most conclusive in setting a clear standard for T2DM medication use in breastfeeding. However, their recommended use of metformin remains “*off‐label”* meaning that diabetic mothers may still be hesitant to use it in breastfeeding. This can be argued for each of the other antidiabetic drugs, especially where clinical studies are totally absent. In some instances, discrepancies occur between guidelines recommendations, for example, The SmPC does not support the use of Glipizide in lactation, whereas the PIL supports “*risk–benefit*” consideration for Pioglitazone and an inconclusive statement for Glipizide use in lactation. Clinical decision making is complex and based on numerous individual factors beyond the question of drug exposure to the breastfed infant and includes careful consideration of the benefits‐risks associated with alternative treatments/medications. Although a clinician can weigh up the proposed risk and benefit to the patient based on the disease itself, disease stage and external patient factors, stratification of infant‐associated risk cannot be accurately quantified given the current pharmacokinetic data available. This may compromise patient‐doctor trust and risk negative health outcomes in both mother and infant. In summary, use of all antidiabetic drugs in lactation remains “*off‐label*”. There is a need to conduct more rigorous clinical studies which capture the subtleties and complexities of individual benefits and risks and/or adopt novel in‐silico techniques such as PBPK modeling to promote consensus among guidelines on the safe and effective use of antidiabetic drugs in lactation.

Prediction of pharmacokinetics using new approach methodologies (NAMs) is highly encouraged in drug development.^51^ The Atkinson & Begg algorithm predicts increased mammary epithelial transfer based on drug properties including molecular weight (< 500 g/mol), protein binding (20–50%), hydrophilicity (LogD 0–< 3), and pKa (5–7).^58^ Among study drugs, sulfonylureas, DPP4 inhibitors, and thiazolidinediones may have higher M:P ratios and theoretical infant risk due to characteristics favoring passive diffusion. Conversely, GLP‐1 RAs (complex structure, high molecular weight), biguanides (hydrophilic, high protein binding), and SGLT2‐I (high protein binding, predominantly ionized) have limited barrier crossing ability.^59^ The Atkinson model assumes bi‐directional passive diffusion driven by concentration gradients and available unbound drug. Acidic drugs like sulfonylureas (Glyburide pKa = 4.32, Glimepiride pKa = 2.23) may experience reverse “ion‐trapping,” with increased clearance back to plasma due to favorable movement to the alkaline plasma compartment. However, these estimations remain empirical and may not reflect clinical observations. Actual drug transfer involves complex drug‐physiological interactions, including genetic variability and transporter/enzyme system maturation. For example, the Atkinson and Begg algorithm predicts Metformin (pKa 11.5, *f*_u,p_ 0.9, LogP ‐1.0) M:P ratio of 2.59, which is 7.6‐fold higher than the measured value of 0.34.^26^ More accurate evidence requires extensive predictive techniques including integration of PBPK modeling techniques with existing algorithms with PBPK techniques.^60^

## CONCLUSION

Literature evaluating safety of T2DM medication in breastfeeding is minimal. Of the literature that does exist, variable methodology has been employed, and guidance is often contradictory or incomplete. Compared with other therapeutic areas, there is a need for rigorous clinical studies and adoption of novel modeling approaches, including PBPK Modeling to generate evidence on breastmilk transfer and infant exposure to T2DM drug therapies.

## FUNDING

This work was supported, in whole or in part, by the Gates Foundation INV052008. The conclusions and opinions expressed in this work are those of the author(s) alone and shall not be attributed to the Foundation. Under the grant conditions of the Foundation, a Creative Commons Attribution 4.0 License has already been assigned to the Author Accepted Manuscript version that might arise from this submission. CW was supported by Wellcome Clinical Research Career Development Fellowship 222075/Z/20/Z and subsequently by NIHR Global Health Research Professorship NIHR304266. The views expressed are those of the author(s) and not necessarily those of the NIHR or the Department of Health and Social Care.

## CONFLICT OF INTEREST STATEMENT

The authors declare no conflicts of interest.

## AUTHOR CONTRIBUTIONS

KR and CW designed the research, KR and JK performed the research, KR, JK, and FWO analyzed the data, and KR, JK, BMO, FWO, and CW wrote the manuscript.

## Supporting information


Data S1
