# Supplementary material for: Maternal‐to‐Infant Transfer of Medications for Type 2 Diabetes Mellitus Via Breastmilk: A Systematic Review of Available Evidence and Clinical Guidelines
Source: Clin Pharmacol Ther. 2026 Feb 11;119(5):1223–34. doi: 10.1002/cpt.70211 (PMC13083373; doi:10.1002/cpt.70211)
Supplement: Supplementary file 1 — Data S1 [file CPT-119-1223-s001.docx]

**Supporting information**

1. **Table 1: ClinPK Checklist score**

| **ClinPK check list item** | Briggs *et. al* (25) | Eyal *et. al.* (26) | Feig DS *et. al.* (30) | Gardiner *et. al.* (29) | Gardiner *et. al.* (27) | Hale *et. al.* (28) | Diab *et. al.* (31) | Moiel *et. al.* (32) |
| --- | --- | --- | --- | --- | --- | --- | --- | --- |
| 1 The title identifies the drug(s) and patient population(s) studied | 1 | 1 | 1 | 1 | 1 | 1 | 1 | 1 |
| 2 The abstract minimally includes the name of the drug(s) studied, the route of administration, the population in whom it was studied, and the results of the primary objective and major clinical pharmacokinetic findings | 1 | 1 | 1 | 1 | 0 | 1 | 1 | 0 |
| 3 Pharmacokinetic data (i.e., absorption, distribution, metabolism, excretion) that are known and relevant to the drugs being studied are described | 1 | 1 | 0 | 1 | 1 | 1 | 1 | 0 |
| 4 An explanation of study rationale is provided | 1 | 1 | 1 | 1 | 1 | 1 | 1 | 1 |
| 5 Specific objectives or hypotheses are provided | 1 | 1 | 1 | 1 | 1 | 1 | 1 | 1 |
| 6 Eligibility criteria of study participants are described | 1 | 1 | 1 | 0 | 1 | 1 | 0 | 1 |
| 7 Co-administration (or lack thereof) of study drug(s) with other potentially interacting drugs or food within this study is described | 0 | 0 | 1 | 1 | 1 | 0 | 1 | 0 |
| 8 Drug preparation and administration characteristics including dose, route, formulation, infusion duration (if applicable) and frequency are described | 0 | 1 | 1 | 1 | 1 | 1 | 1 | 1 |
| 9 Body fluid or tissue sampling (timing, frequency and storage) for quantitative drug measurement is described | 1 | 1 | 1 | 1 | 1 | 1 | 1 | 1 |
| 10 Validation of quantitative bioanalytical methods used in the study is referenced or described if applicable | 1 | 1 | 1 | 1 | 0 | 1 | 1 | 0 |
| 11 Pharmacokinetic modelling methods and software used are described, including assumptions made regarding the number of compartments and order of kinetics (zero, first or mixed order) | 0 | 1 | 1 | 1 | 1 | 1 | 1 | 0 |
| 12 For population pharmacokinetic studies, covariates incorporated into pharmacokinetic models are identified and described | N/A | N/A | N/A | N/A | N/A | N/A | N/A | N/A |
| 13 Formulas for calculated variable (such as creatinine clearance, body surface area, AUC [area under the curve] and adjusted body weight) are provided or referenced | 1 | 1 | 1 | 1 | 1 | 1 | 1 | 0 |
| 14 The specific body weight used in drug dosing and pharmacokinetic calculations is reported i.e. ideal body weight vs actual body weight vs adjusted body weight | 1 | 1 | 1 | 1 | 1 | 1 | 1 | 0 |
| 15 Statistical methods including software used are described | 0 | 1 | 0 | 0 | 0 | 1 | 0 | 0 |
| 16 Study withdrawals or subjects lost to follow up (or lack thereof) are reported | 1 | 1 | 1 | 0 | N/A | 0 | N/A | 0 |
| 17 Quantification of missing or excluded data is provided if applicable | 1 | 1 | 1 | 1 | 0 | 0 | 0 | 0 |
| 18 All relevant variables that may explain inter- and intra-patient pharmacokinetic variability (including: age, sex, end-organ function, ethnicity, weight or BMI [body mass index], health status or severity of illness and pertinent co-morbidities) are provided with appropriate measures of variance | 1 | 1 | 1 | 1 | 1 | 1 | 1 | 0 |
| 19 Results of pharmacokinetic analyses are reported with appropriate measures of precision (such as range or 95% confidence intervals) | 1 | 1 | 1 | 1 | 1 | 1 | 1 | N/A |
| 20 Studies in patients receiving extracorporeal drug removal (i.e. dialysis) should report the mode of drug removal, type of filters used, duration of therapy and relevant flow rates | N/A | N/A | N/A | N/A | N/A | N/A | N/A | N/A |
| 21 In studies of drug bioavailability comparing two formulations of the same drug, F (bioavailability), AUC, Cmax (maximal concentration) and Tmax (time to maximal concentration) should be reported | N/A | N/A | N/A | N/A | N/A | N/A | N/A | N/A |
| 22 Study limitations describing potential sources of bias and imprecision where relevant should be described | 0 | 1 | 1 | 1 | 0 | 0 | 1 | 0 |
| 23 The relevance of study findings (applicability, external validity) is described | 1  1 | 1 | 1 | 1 | 1 | 1 | 1 | 0 |
| 24 Funding sources and conflicts of interest for the authors are also disclosed | 1 | 1 | 1 | 1 | 0 | 1 | 1 | 0 |
| **Total, ClinPK score / Score of relevant ClinPK items** | 14/21 | 15/21 | 17/21 | 16/21 | 14/20 | 14/21 | 16/20 | 9/20 |
| **Percentage Score (%)** | 66.7 | 71.4 | 81 | 76.2 | 70 | 66.7 | 80 | 45 |

1. **Table 2:** Summary of available clinical evidence on the use of anti-diabetic drugs during breastfeeding

|  | Royal Australian College of General Practitioners (RACGP) (54) | Derbyshire Joint Area Prescribing Committee (JAPC) – 2022 (55) | Northeast London guidelines  Type 2 Diabetes: Preventing complications Guideline for the Management of Type 2 Diabetes (56) | SmPC from EMC | PIL form EMC | NICE Guidelines- BNF drug summary (57) | LactMed (58) | WHO (1994)(59) | Organization of Teratology Information Specialists (OTIS) (60) |
| --- | --- | --- | --- | --- | --- | --- | --- | --- | --- |
| Metformin | Y (6) | - | - | N (3) | N | Y | Y (6) | N (4) | R (6) |
| Dapagliflozin | - | N (4) | N (4) | N (1) (2) | N (4) | N | N (2, 3, 5) | - | - |
| Empagliflozin | - | N(4) | N ( 4) | N (1, 2, 3) | N (4) | N | N (2, 3, 5) | - | - |
| Canagliflozin | - | N(4) | N (4) | N (1, 2, 4) | N | N | N (2, 3, 5) | - | - |
| Ertugliflozin | - | N(4) | N (4) | N (1, 2, 3) | N (4) | N | N (2, 3, 5) | - | - |
| Alogliptin | - | - | - | R (1, 2, 4, ) | N (3) | N | A (3) | - | - |
| Saxagliptin | - | - | - | R (1, 2, 4, ) | N (4) | N | A (3, 5) | - | - |
| Vildagliptin | - | - | - | N (1, 4) | N (4) | N | - | - | - |
| Sitagliptin | - | - | - | N (1, 4) | N (4) | N | A (3, 5) | - | - |
| Linagliptin | - | - | - | R (1, 2, 4, ) | R (4) | N | A (3, 5) | - | - |
| Tolbutamide | - | - | - | N (1, 2, 4) | N | N | I (3) | N(4) | - |
| Glyburide | - | - | - | Y | Y | N | - | N(4) | - |
| Glimepiride | - | - | - | N (1, 4) | N | N | A (3) | N(4) | - |
| Gliclazide | - | - | - | N (2, 4) | N | N | - | N(4) | - |
| Glipizide | - | - | - | N (3) | I | N | A (3) | N(4) | - |
| Semaglutide | - | N (4) | - | N (1, 2) | N (4) | N | - | - | R (6) |
| Exenatide | - | N (4) | - | N (4) | N (4) | N | - | - | - |
| Liraglutide | - | N (4) | - | N (1, 3, 4) | N (4) | N | - | - | - |
| Tirzepatide | - | N (4) | - | R (2, 4) | R (2, 4) | N | - | - | - |
| Pioglitazone | - | - | - | N (1, 4) | R | N | A (3, 5) | - | - |

**^Reasons for Guideline decision: 1:^** ^Trialed in animals^**^; 2:^** ^Potential risk in nursing infant^**^; 3:^** ^Limited data/ ‘relevant published data not found as of publication data’^**^; 4:^** ^’ It is unknown’^**^; 5:^** ^Relation to pharmacological properties of drug (half-life, plasma protein binding);^ **^6:^** ^Studies found on transfer of drug in breastfeeding^

1. **Table 3: Summary of available clinical evidence**

| Drug | Royal Australian College of General Practitioners (RACGP) | Derbyshire Joint Area Prescribing Committee (JAPC) - 2022 | Northeast London  Type 2 Diabetes: Preventing complications Guideline for the Management of Type 2 Diabetes | SmPC from EMC | PIL form EMC | WHO-EM/DIN6/E/G, 1994  Management Of Diabetes Mellitus Standards of Care and Clinical Practice Guidelines | NICE Guidelines- BNF drug summary | American Association of Clinical Endocrinologists (AACE) | LactMed | PubMed |
| --- | --- | --- | --- | --- | --- | --- | --- | --- | --- | --- |
| Metformin | Metformin may be continued while breastfeeding with minimal effect on the baby.26 Breastfeeding may alter glucose levels, so glycemic monitoring, oral medications and insulin need careful review during breastfeeding to minimize the risk of hypoglycemia |  | Contraindications: Pregnancy and breastfeeding (counsel patients of childbearing potential on the risks of taking a SGLT-2i during pregnancy)  Before starting this medication tell your doctor/diabetes specialist nurse if you are Planning or are pregnant or breastfeeding. | ’Metformin is excreted into human breast milk. No adverse effects were observed in breastfed newborns/infants. However, as only limited data are available, breastfeeding is not recommended during metformin treatment. A decision should be made whether to discontinue nursing.  or to discontinue metformin, considering the importance of the compound to the mother.’’ | ’Do not take if you are pregnant or breastfeeding. Ask your doctor or pharmacist before taking any medication.’’ | Both **SUs** and Biguanides (e.g. metformin) should not be used during pregnancy or breast-feeding | [Metformin hydrochloride](https://bnf.nice.org.uk/drugs/metformin-hydrochloride/) can be continued immediately after birth and during breast-feeding for those with pre-existing [Type 2 diabetes](https://bnf.nice.org.uk/treatment-summaries/type-2-diabetes/). Note that this is an off-label use of metformin.’ |  | ‘’Data from well-conducted studies indicate that metformin levels in milk are low, and infants would receive less than 0.5% of their mother's weight adjusted dosage. Milk metformin levels are relatively constant during maternal metformin use, so timing of breastfeeding with respect to the administration times is of little benefit. Although the dose in milk is low, metformin is sometimes detectable in low levels in the serum of breastfed infants. One sizeable prospective study found no adverse effects in breastfed infants. Metformin should be used with caution while nursing newborn and premature infants and those with renal impairment.’’ | 5 relevant papers found. |
| Dapagliflozin |  | Avoid use of SGLT2I in the following situations: Pregnant, breastfeeding, female in the child-bearing years and sexually active without contraception |  | ‘’Unknown whether dapagliflozin and/or its metabolites are excreted in human milk. Available pharmacodynamic/toxicological data in animals have shown excretion of dapagliflozin/metabolites in milk, as well as pharmacologically mediated effects in nursing offspring. A risk to the newborns/infants cannot be excluded. Dapagliflozin should not be used while breast-feeding.’’ | ’Do not use if you are breast-feeding. It is not known if this medicine passes into human breast milk.’’ |  | All other antidiabetic drugs should be avoided while breast-feeding. |  | ‘’Relevant published information was not found as of the revision date.’’ ‘’No information is available on the clinical use of dapagliflozin during breastfeeding. Dapagliflozin is an uncharged molecule that is 91% protein bound in plasma, so it is unlikely to pass into breast milk in clinically important amounts. The manufacturer does not recommend dapagliflozin during breastfeeding because of a theoretical risk to the infant's developing kidney.’’ |  |
| Empagliflozin |  |  |  | ’No data in humans are available on excretion of empagliflozin into milk. Available toxicological data in animals have shown excretion of empagliflozin in milk. A risk to the newborns/infants cannot be excluded. Should not be used during breast-feeding.’ | ’Do not use if you are breast-feeding. It is not known if it passes into human breast milk.’’ |  | All other antidiabetic drugs should be avoided while breast-feeding. |  | ‘’Relevant published information was not found as of the revision date.’’ ‘’No information is available on the clinical use of Empagliflozin during breastfeeding. Empagliflozin is an uncharged molecule that is 86% protein bound in plasma, so it is unlikely to pass into breast milk in clinically important amounts. The manufacturer does not recommend empagliflozin during breastfeeding because of a theoretical risk to the infant's developing kidney.’’ |  |
| Canagliflozin |  |  |  | ‘’Unknown whether canagliflozin and/or its metabolites are excreted in human milk. Available pharmacodynamic/toxicological data in animals have shown excretion of canagliflozin/metabolites in milk, as well as pharmacologically mediated effects in breast-feeding offspring and juvenile rats exposed to canagliflozin. A risk to newborns/infants cannot be excluded. Canagliflozin should not be used during breastfeeding.’’ | ’You should not take this medicine if you are breast-feeding. Talk to your doctor about whether to stop taking this medicine or to stop breast-feeding.’’ |  | All other antidiabetic drugs should be avoided while breast-feeding. |  | ‘’Relevant published information was not found as of the revision date.’’ ‘’No information is available on the clinical use of canagliflozin during breastfeeding. Canagliflozin is an uncharged molecule that is 99% protein bound in plasma, so it is unlikely to pass into breast milk in clinically important the manufacturer does not recommend canagliflozin during breastfeeding because of a theoretical risk to the infant's developing kidney.’’ amounts. |  |
| Ertugliflozin |  |  |  | ‘’There is no information regarding the presence of ertugliflozin in human milk, the effects on the breast-fed infant, or the effects on milk production. Ertugliflozin is present in the milk of lactating rats and caused effects in the offspring of lactating rats. Pharmacologically mediated effects were observed in juvenile. Since human kidney maturation occurs in utero and during the first 2 years of life when exposure from breastfeeding may occur, a risk to newborns/infants cannot be excluded. Should not be used during breast-feeding.’’ | ’It is not known if drug passes into breast milk. Talk with your doctor about the best way to feed your baby if you take this medication. ‘’Do not use if you are breast-feeding.’’ |  | All other antidiabetic drugs should be avoided while breast-feeding. |  | ‘’Relevant published information was not found as of the revision date.’’ ‘’No information is available on the clinical use of Ertugliflozin during breastfeeding. Ertugliflozin is 94% protein bound in plasma, so it is unlikely to pass into breast milk in clinically important amounts. The manufacturer does not recommend ertugliflozin during breastfeeding because of a theoretical risk to the infant's developing kidney. An alternate drug may be preferred, especially while nursing a newborn or preterm infant.’’ |  |
| Alogliptin |  |  |  | ‘’Unknown whether alogliptin is excreted in human milk. Animal studies have shown excretion of alogliptin in milk. A risk to the suckling child cannot be excluded. A decision on whether to discontinue breastfeeding or to discontinue alogliptin therapy should be made considering the benefit of breast-feeding for the child and the benefit of alogliptin therapy for the woman.’’ ‘’Studies in lactating rats indicate that alogliptin is excreted in milk. No alogliptin.  elated effects were observed in juvenile rats following repeat-dose administration for 4 and 8 weeks.’’ | ‘’There is no experience of use in pregnant women or during breast-feeding. Should not be used during pregnancy or breastfeeding. Your doctor will help you to decide whether to continue breast-feeding or to continue use.’’ |  | All other antidiabetic drugs should be avoided while breast-feeding. |  | ’Relevant published information was not found as of the revision date.’’ ‘’No information is available on the clinical use of alogliptin during breastfeeding. An alternate drug may be preferred, especially while nursing a newborn or preterm infant. Monitoring of the breastfed infant's blood glucose is advisable during maternal therapy with alogliptin.’’ |  |
| Saxagliptin |  |  |  | ‘’Unknown whether saxagliptin is excreted in human breast milk. Animal studies have shown excretion of saxagliptin and/or metabolite in milk. A risk to the suckling child cannot be excluded. A decision must be made whether to discontinue breast-feeding or to discontinue therapy considering the benefit of breast-feeding for the child and the benefit of therapy to the woman.’’ | ’Talk to your doctor if you want to breast feed while taking this medicine. It is not known if it passes into human breast milk. You should not take this medicine if you are breast-feeding or plan to breast-feed. |  | All other antidiabetic drugs should be avoided while breast-feeding. |  | ’Relevant published information was not found as of the revision date.’’ ‘’No information is available on the clinical use of Saxagliptin during breastfeeding. Saxagliptin has a shorter half-life than the other dipeptidyl peptidase IV inhibitors, so it might be a better choice among drugs in this class for nursing mothers. Monitoring of the breastfed infant's blood glucose is advisable during maternal therapy with Saxagliptin. However, an alternate drug may be preferred, especially while nursing a newborn or preterm infant.’’ |  |
| Vildagliptin |  |  |  | ’Unknown whether Vildagliptin is excreted in human milk. Animal studies have shown excretion of Vildagliptin in milk. Should not be used during breast-feeding.’’ | ’It is not known if it passes into breast milk. You should not use if you are breast-feeding or plan to breast-feed.’’ |  | All other antidiabetic drugs should be avoided while breast-feeding. |  |  |  |
| Sitagliptin |  |  |  | ‘’Unknown whether sitagliptin is excreted in human breast milk. Animal studies have shown excretion of sitagliptin in breast milk. Sitagliptin should not be used during breastfeeding.’’ | ’It is not known if this medicine passes into breast milk. You should not take this medicine if you are breastfeeding or plan to breast-feed.’ |  | All other antidiabetic drugs should be avoided while breast-feeding. |  | ’Relevant published information was not found as of the revision date.’’ ‘’No information is available on the clinical use of linagliptin during breastfeeding. Linagliptin's plasma protein binding ranges from 80% to over 99%, so it is unlikely to pass into breast milk in clinically important amounts and might be a better choice among drugs in this class for nursing mothers. However, an alternate drug may be preferred, especially while nursing a newborn or preterm infant. ‘’ |  |
| Linagliptin |  |  |  | ‘’Available pharmacokinetic data in animals have shown excretion of linagliptin/metabolites in milk. A risk to the breast-fed child cannot be excluded. A decision must be made whether to discontinue breast-feeding or to discontinue/abstain from linagliptin therapy considering the benefit of breastfeeding for the child and the benefit of therapy for the woman.’’ | ‘’It is not known if it passes into human breast milk. A decision must be made by your doctor whether to discontinue breastfeeding or to discontinue/abstain from use.’’ |  | All other antidiabetic drugs should be avoided while breast-feeding. |  | ’Relevant published information was not found as of the revision date.’’ ‘’No information is available on the clinical use of linagliptin during breastfeeding. Linagliptin's plasma binding ranges from 80% to over 99%, so it is unlikely to pass into breast milk in clinically important amounts and might be a better choice among drugs in this class for nursing mothers. However, an alternate drug may be preferred, especially while nursing a newborn or preterm infant. ‘’ |  |
| Tolbutamide |  |  |  | ‘’Tolbutamide has been detected in breast milk in small quantities. The effect on the neonate is unknown but there is a theoretical risk of hypoglycemia. Breast-feeding is best avoided in mothers taking tolbutamide.’’ | ‘’Do not take tolbutamide if you are breastfeeding.’’ | Both **SUs** and Biguanides (e.g. metformin) should not be used during pregnancy or breast-feeding | All other antidiabetic drugs should be avoided while breast-feeding. |  | ‘’Relevant published information was not found as of the revision date.’’ ‘’Tolbutamide is no longer marketed in the United States.  It is excreted into breast milk in small amounts that should cause no harm to the breastfed infant. ‘’ | 1 relevant paper found |
| Glyburide |  |  |  | ‘Breast-feeding seems to be compatible, but as a precautious measure monitoring of the fully breast-fed infant's blood sugar level is advisable.’ | ‘Breast-feeding seems to be compatible, but as a precautious measure monitoring of the fully breast-fed infant's blood sugar level is advisable.’ | Both **SUs** and Biguanides (e.g. metformin) should not be used during pregnancy or breast-feeding | All other antidiabetic drugs should be avoided while breast-feeding. |  |  |  |
| Glimepiride |  |  |  | ’The excretion in human milk is unknown. Glimepiride is excreted in rat milk. As other sulfonylureas are excreted in human milk and because there is a risk of hypoglycemia in nursing infants, breast-feeding is advised against during treatment with Glimepiride.’’ | ‘’Glimepiride may pass into breast milk. Glimepiride should not be taken during breastfeeding. If you are pregnant or breastfeeding, think you may be pregnant or are planning to have a baby, ask your doctor or r pharmacist for advice before taking this medicine.’’ | Both **SUs** and Biguanides (e.g. metformin) should not be used during pregnancy or breast-feeding | All other antidiabetic drugs should be avoided while breast-feeding. |  | ‘’Relevant published information was not found as of the revision date.’’ ‘’Because no information is available on the use of Glimepiride during breastfeeding, an alternate drug may be preferred, especially while nursing a newborn or preterm infant.’’ |  |
| Gliclazide, |  |  |  | ’It is not known whether gliclazide or its metabolites are excreted in human milk. Given the risk of neonatal hypoglycemia, this medicinal product is therefore contra indicated in breast-feeding mothers. A risk to the newborns/infants cannot be excluded.’’ | Do not take if you are breastfeeding.’’ | Both **SUs** and Biguanides (e.g. metformin) should not be used during pregnancy or breast-feeding | All other antidiabetic drugs should be avoided while breast-feeding. |  |  |  |
| Glyburide |  |  |  |  |  | Both **SUs** and Biguanides (e.g. metformin) should not be used during pregnancy or breast-feeding | All other antidiabetic drugs should be avoided while breast-feeding. |  | Relevant published information was not found as of the revision date | 1 relevant paper found. |
| Glipizide |  |  |  | ‘’No data are available on secretion into breast milk. Therefore, glipizide is contraindicated in lactation.’’ | ’If you are pregnant or breast-feeding, think you may be pregnant or are planning to have a baby, ask your doctor or pharmacist for advice before taking this medicine.’’ | Both **SUs** and Biguanides (e.g. metformin) should not be used during pregnancy or breast-feeding | All other antidiabetic drugs should be avoided while breast-feeding. |  | ’Relevant published information was not found as of the revision date.’’ ‘’Limited data indicate that the levels of glipizide in milk are low. However, an alternate drug for which there is more information may be preferred, especially while nursing a newborn or preterm infant. ‘’ | 1 relevant paper found. |
| Semaglutide |  | Contraindicated in Type 1 diabetes, pregnancy and breastfeeding |  | ‘In lactating rats, semaglutide was excreted in milk. As a risk to a breast-fed child cannot be excluded, semaglutide should not be used during breast-feeding.’ | ‘’ Do not use this medicine if you are breast-feeding, as it is unknown if it passes into breast milk.’’ |  | All other antidiabetic drugs should be avoided while breast-feeding. |  |  | 1 relevant paper found |
| Exenatide |  | Contraindicated in breast-feeding |  | It is unknown whether exenatide is excreted in human milk. Prolonged release exenatide should not be used during breast-feeding. | ‘’ It is not known if exenatide passes into your milk. You should not use this medicine while breast-feeding. If you are pregnant or breast-feeding, think you may be pregnant, or are planning to have a baby, ask your doctor or pharmacist for advice before taking this medicine.’’ |  | All other antidiabetic drugs should be avoided while breast-feeding. |  |  |  |
| Liraglutide |  | Contraindicated in breast-feeding |  | It is not known whether liraglutide is excreted in human milk. Animal studies have shown that the transfer of liraglutide and metabolites of close structural relationship into milk is low. Non-clinical studies have shown a treatment-related reduction of neonatal growth in suckling rat pups (see section 5.3). Because of lack of experience, BIOLIDE injection should not be used during breast-feeding. | It is not known if Diavic® passes into breast milk, therefore do not use this medicine if you are breast- feeding. |  | All other antidiabetic drugs should be avoided while breast-feeding. |  |  |  |
| Tirzepatide |  |  |  | It is unknown whether tirzepatide is excreted in human milk. A risk to the newborn/infant cannot be excluded.  A decision must be made whether to discontinue breast-feeding or to discontinue/abstain from tirzepatide therapy considering the benefit of breast-feeding for the child and the benefit of therapy for the woman. | It is unknown whether tirzepatide passes into breast milk. A risk to newborns/infants cannot be ruled out. If you are breast-feeding or are planning to breast-feed, talk to your doctor before using this medicine. You and your doctor should decide if you should stop breast-feeding or delay using Mounjaro®. |  | All other antidiabetic drugs should be avoided while breast-feeding. |  |  |  |
| Pioglitazone |  |  |  | ’Pioglitazone has been shown to be present in the milk of lactating rats. It is not known whether pioglitazone is secreted in human milk. Therefore, pioglitazone should not be administered to breastfeeding women.’’ | ‘’Tell your doctor if you are breast-feeding or if you are planning to breast-feed your baby. Your doctor will advise not to discontinue this medicine.’’ |  | All other antidiabetic drugs should be avoided while breast-feeding. |  | ’Relevant published information was not found as of the revision date.’’ ‘’No information is available on the clinical use of pioglitazone during breastfeeding.  Pioglitazones is over 99% protein bound in plasma, so it is unlikely to pass into breast milk in clinically important amounts. However, an alternate drug may be preferred, especially while nursing a newborn or preterm infant.’’ |  |

**4.0 Table 4: Summary of drug-specific outcomes and safety assessment based on U.S FDA criteria**

| **Drug** | **Study Details** | **Detection Results** | **Safety Assessment** |
| --- | --- | --- | --- |
| **Glyburide** | -Study: Feig et al. (37)  -Design: Single dose, steady state sampling over 8-hour interval.  -Population: Breastfeeding women with T2DM  -Postpartum period: not explicitly stated. | -Undetectable in both plasma and breast milk  -Theoretical RID: 28% of maternal weight normalized dose  -Based on LLOQ 0.08 µg/ml and 150mL/kg/day milk intake | -Above 10% FDA safety threshold (theoretical)  -Clinical evidence indicates "safe" in lactation  -Theoretical estimates less reliable than clinical data |
| **Glipizide** | -Study: Feig et al. (37)  -Design: Single dose, steady state sampling over 8-hour interval  -Population: Breast-feeding women with T2DM  -Post-partum period:5-8 days | -Undetectable in breast milk  -Detectable in maternal plasma  -Theoretical RID: 27% of maternal weight-normalized dose  -Based on LLOQ 0.08 µg/ml and 150mL/kg/day milk intake | -Above 10% FDA safety threshold (theoretical)  - Clinical evidence indicates "safe" in lactation  - Theoretical estimates less reliable than clinical data |
| **Semaglutide** | -Study: Diab et al. (38)  -Design: ‘’Milk-only’’ study using biorepository samples  -Population: multi-racial lactating mothers (>6 months postpartum) treated for obesity  -sampling ≥ 3 months administrations of same dose (mean 0.56mg/week) | -Undetectable in breast milk samples  -Theoretical weekly accumulation factor: 2.2  - Simulated RIDD: 1.26% of weekly maternal dose  -Based on 0.15L/kg/day milk consumption | -Below 10% FDA safety threshold  -No reported adverse effects in infants  -Deemed "safe" for mean 3-week exposure duration  - Clinical evidence supports safety |

Key Notes

- LLOQ = Lowest Limit of Quantification

- RID = Relative Infant Dose

- RIDD = Relative Infant Daily Dose

- FDA safety threshold: RID <10% generally considered safe

**5.0 Table 5: PRISMA Checklist**

| **Section and Topic** | **Item #** | **Checklist item** | **Location where item is reported** |
| --- | --- | --- | --- |
| **TITLE** | | |  |
| Title | 1 | Identify the report as a systematic review. | 1 |
| **ABSTRACT (SUMMARY)** | | |  |
| Abstract | 2 | See the PRISMA 2020 for Abstracts checklist. | 1 |
| **INTRODUCTION** | | |  |
| Rationale | 3 | Describe the rationale for the review in the context of existing knowledge. | 2 |
| Objectives | 4 | Provide an explicit statement of the objective(s) or question(s) the review addresses. | 2 |
| **METHODS (DESIGN)** | | |  |
| Eligibility criteria | 5 | Specify the inclusion and exclusion criteria for the review and how studies were grouped for the syntheses. | 3 |
| Information sources | 6 | Specify all databases, registers, websites, organisations, reference lists and other sources searched or consulted to identify studies. Specify the date when each source was last searched or consulted. | 3 |
| Search strategy | 7 | Present the full search strategies for all databases, registers and websites, including any filters and limits used. | 3 |
| Selection process | 8 | Specify the methods used to decide whether a study met the inclusion criteria of the review, including how many reviewers screened each record and each report retrieved, whether they worked independently, and if applicable, details of automation tools used in the process. | 3 |
| Data collection process | 9 | Specify the methods used to collect data from reports, including how many reviewers collected data from each report, whether they worked independently, any processes for obtaining or confirming data from study investigators, and if applicable, details of automation tools used in the process. | 3 |
| Data items | 10a | List and define all outcomes for which data were sought. Specify whether all results that were compatible with each outcome domain in each study were sought (e.g. for all measures, time points, analyses), and if not, the methods used to decide which results to collect. | 3 |
|  | 10b | List and define all other variables for which data were sought (e.g. participant and intervention characteristics, funding sources). Describe any assumptions made about any missing or unclear information. | 3 |
| Study risk of bias assessment | 11 | Specify the methods used to assess risk of bias in the included studies, including details of the tool(s) used, how many reviewers assessed each study and whether they worked independently, and if applicable, details of automation tools used in the process. | 4 |
| Effect measures | 12 | Specify for each outcome the effect measure(s) (e.g. risk ratio, mean difference) used in the synthesis or presentation of results. | 4 |
| Synthesis methods | 13a | Describe the processes used to decide which studies were eligible for each synthesis (e.g. tabulating the study intervention characteristics and comparing against the planned groups for each synthesis (item #5)). | 4 |
|  | 13b | Describe any methods required to prepare the data for presentation or synthesis, such as handling of missing summary statistics, or data conversions. | 4 |
|  | 13c | Describe any methods used to tabulate or visually display results of individual studies and syntheses. | 4 |
|  | 13d | Describe any methods used to synthesize results and provide a rationale for the choice(s). If meta-analysis was performed, describe the model(s), method(s) to identify the presence and extent of statistical heterogeneity, and software package(s) used. | N/A |
|  | 13e | Describe any methods used to explore possible causes of heterogeneity among study results (e.g. subgroup analysis, meta-regression). | N/A |
|  | 13f | Describe any sensitivity analyses conducted to assess robustness of the synthesized results. | N/A |
| Reporting bias assessment | 14 | Describe any methods used to assess risk of bias due to missing results in a synthesis (arising from reporting biases). | N/A |
| Certainty assessment | 15 | Describe any methods used to assess certainty (or confidence) in the body of evidence for an outcome. | N/A |
| **RESULTS** | | |  |
| Study selection | 16a | Describe the results of the search and selection process, from the number of records identified in the search to the number of studies included in the review, ideally using a flow diagram. | 4 |
|  | 16b | Cite studies that might appear to meet the inclusion criteria, but which were excluded, and explain why they were excluded. | 4 |
| Study characteristics | 17 | Cite each included study and present its characteristics. | 5 |
| Risk of bias in studies | 18 | Present assessments of risk of bias for each included study. | N/A |
| Results of individual studies | 19 | For all outcomes, present, for each study: (a) summary statistics for each group (where appropriate) and (b) an effect estimates and its precision (e.g. confidence/credible interval), ideally using structured tables or plots. | 5 & 6 |
| Results of syntheses | 20a | For each synthesis, briefly summarise the characteristics and risk of bias among contributing studies. | 5&6 |
|  | 20b | Present results of all statistical syntheses conducted. If meta-analysis was done, present for each the summary estimate and its precision (e.g. confidence/credible interval) and measures of statistical heterogeneity. If comparing groups, describe the direction of the effect. | 5&6 |
|  | 20c | Present results of all investigations of possible causes of heterogeneity among study results. | N/A |
|  | 20d | Present results of all sensitivity analyses conducted to assess the robustness of the synthesized results. | N/A |
| Reporting biases | 21 | Present assessments of risk of bias due to missing results (arising from reporting biases) for each synthesis assessed. | N/A |
| Certainty of evidence | 22 | Present assessments of certainty (or confidence) in the body of evidence for each outcome assessed. | N/A |
| **DISCUSSION** | | |  |
| Discussion | 23a | Provide a general interpretation of the results in the context of other evidence. | 6 |
|  | 23b | Discuss any limitations of the evidence included in the review. | 7 |
|  | 23c | Discuss any limitations of the review processes used. | N/A |
|  | 23d | Discuss implications of the results for practice, policy, and future research. | 9 |
| **OTHER INFORMATION** | | |  |
| Registration and protocol | 24a | Provide registration information for the review, including register name and registration number, or state that the review was not registered. | 2 |
|  | 24b | Indicate where the review protocol can be accessed, or state that a protocol was not prepared. | 2 |
|  | 24c | Describe and explain any amendments to information provided at registration or in the protocol. | N/A |
| Support | 25 | Describe sources of financial or non-financial support for the review, and the role of the funders or sponsors in the review. | 9 |
| Competing interests | 26 | Declare any competing interests of review authors. | 9 |
| Availability of data, code and other materials | 27 | Report which of the following are publicly available and where they can be found: template data collection forms; data extracted from included studies; data used for all analyses; analytic code; any other materials used in the review. | N/A |

1. **Table 6: Literature search results**

| **S/N** | **Drug** | **Sources** | **Initial screen of retrieved articles** | **Included at initial title screening** | **Included at abstract screening** | **Included at full text screening** | **Inclusion in the review** | **Duplicates** | **Number of studies analyzed** |
| --- | --- | --- | --- | --- | --- | --- | --- | --- | --- |
|  | Metformin | Google scholar | 11, 000 | 742 | 7 | 3 | 3 | 8 | 5 |
|  |  | PubMed | 444 | 4 (Not mention of Metformin) | 3 | 3 | 3 |  |  |
|  |  | Scopus | 8, 189 | 45 (Not drug of metformin in title and abstract) | 19 | 13 | 4 |  |  |
|  |  | LactMed | 10 | 7 | 4 | 3 | 3 |  |  |
|  | Tolbutamide | Google scholar | 11, 000 | 6 | 0 | 0 | 0 | 0 | 1 |
|  |  | PubMed | 444 | 0 | 0 | 0 | 0 |  |  |
|  |  | Scopus | 8, 189 | 0 | 0 | 0 | 0 |  |  |
|  |  | LactMed | 3 | 3 | 2 | 1 | 1 |  |  |
|  | Glyburide | Google scholar | 11, 000 | 339 | 5 | 1 | 1 | 3 | 1 |
|  |  | PubMed | 444 | 1 | 1 | 1 | 1 |  |  |
|  |  | Scopus | 8, 189 | 12 (Not drug of metformin in title and abstract) | 1 | 1 | 1 |  |  |
|  |  | LactMed | 5 | 5 | 2 | 1 | 1 |  |  |
|  | Glipizide | Google scholar | 11, 000 | 132 | 2 | 1 | 1 | 3 | 1 |
|  |  | PubMed | 444 | 1 | 1 | 1 | 1 |  |  |
|  |  | Scopus | 8, 189 | 2 (Not drug of metformin in title and abstract) | 1 (Inaccessible abstract) | 1 | 1 |  |  |
|  |  | LactMed | 3 | 3 | 1 (non-lactational studies) | 1 | 1 |  |  |
|  | Semaglutide | Google scholar | 11, 000 | 54 | 1 | 1 | 1 | 1 | 1 |
|  |  | PubMed | 444 | 0 | 0 | 0 | 0 |  |  |
|  |  | Scopus | 8, 189 | 6 (Not drug of metformin in title and abstract) | 1 (Duplicates) | 1 | 1 |  |  |
|  |  | LactMed | 0 | 0 | 0 | 0 | 0 |  |  |

**Search terms:** For each drug, a search strategy was conducted by replacing “*drug*” with the drug name: (Drug) AND (blood OR serum OR plasma OR human) AND (breast) AND (milk) AND (postpart* OR breastfeed* OR lactat*) AND (Pharmacokinet*)

- Initial retrieved (**19, 654**): Online search engines = Scholar (11, 000), Pubmed (444), Scopus (8, 189), Database = LactMed (21)
- Included in Abstract screening (1, 352): Online search engines = Scholar (1, 273), Pubmed (6), Scopus (65), Database = LactMed (18)
- Included in full-text screening (**51**): Online search engines = Scholar (15), Pubmed (5), Scopus (22), Database = LactMed (9)
  - Excluded due to irretrievable full-text article = 09
- Included in full-text article review = 42
  - Excluded due to reasons in Fig. 1 = 34
- Included in Review (**8**): Online search engines = Scholar (6), Pubmed (5), Scopus (16), Database = LactMed (6)
- One study is shared between Glyburide and Glipizide, thus only 8 studies included in the study.
